# ALDOC and PGK1 coordinately induce glucose metabolism reprogramming and promote development of colorectal cancer

**DOI:** 10.1186/s10020-025-01252-z

**Published:** 2025-06-15

**Authors:** Liyong Huang, Yixin Tong, Xu Li, Wei Wang, Zhen Wang, Bingchen Chen, Jifu E., Ruzhen Zhou, Hantao Wang, Jinghu He

**Affiliations:** 1 Department of Colorectal Surgery, Fudan University Shanghai Cancer Center, No.270 Dongan Road, Xuhui District, Shanghai, 200032 China; 2 Department of GI Surgery, Tongji Hospital of Tongji Medical College of Huazhong University of Science and Technology, Wuhan, Hubei China; 3 Department of Colorectal Surgery, The First Affiliated Hospital of Naval Medical University, No.168, Changhai Road, Shanghai, 200433 China; 4 Department of General Surgery, Shanghai 411 Hospital, RongTong Medical Healthcare Group Co. Ltd., Shanghai, 200081 China

**Keywords:** Colorectal cancer, ALDOC, PGK1, Glycolysis, Molecular mechanism

## Abstract

Colorectal cancer (CRC) remains a significant health challenge globally, demanding a comprehensive understanding of its molecular underpinnings for effective management. In this study, we investigated the role of Aldolase C (ALDOC), a glycolytic enzyme, in CRC pathogenesis. Transcriptomic analysis of CRC tissues from The Cancer Genome Atlas (TCGA) revealed a substantial upregulation of ALDOC, correlating with adverse clinical outcomes. Immunohistochemical (IHC) staining of locally collected patient-derived tissues corroborated these findings, demonstrating elevated ALDOC expression in tumor tissues, particularly in advanced stages. Functional studies elucidated the regulatory role of ALDOC in CRC cell phenotypes. ALDOC knockdown significantly inhibited cell proliferation, induced apoptosis, arrested cell cycle progression, and suppressed cell migration in vitro. Moreover, in vivo studies using xenograft models confirmed that ALDOC knockdown attenuated tumor growth. Mechanistically, ALDOC was found to interact with hypoxia-inducible factor 1 alpha (HIF1A) and enhance its transcriptional activity on phosphoglycerate kinase 1 (PGK1), a key glycolytic enzyme. Dual-luciferase reporter assays and chromatin immunoprecipitation experiments validated the ALDOC-mediated transcriptional activation of PGK1. Further functional rescue experiments revealed a synergistic interplay between ALDOC and PGK1 in regulating CRC cell phenotypes. Additionally, ALDOC was implicated in promoting aerobic glycolysis in CRC cells, potentially through PGK1 regulation. Collectively, our findings unveil ALDOC as a critical regulator of CRC pathogenesis, offering insights into its potential as a therapeutic target and highlighting the ALDOC/PGK1 axis as a promising avenue for further investigation in CRC.

**Supplementary Information:**

The online version contains supplementary material available at 10.1186/s10020-025-01252-z.

## Introduction

Colorectal cancer is one of the most common malignant tumors of the digestive system and is one of the leading causes of tumor-related death (Ferlay et al. 2021; Siegel et al. 2022). Colorectal cancer is characterized by high morbidity and mortality, making it one of the major threats to human health worldwide. In recent years, with the rapid development of minimally invasive surgery and precision surgery concept, the generation of treatment strategies of surgery combined with other treatment methods, and the emergence of molecular targeted therapy/immunotherapy, the prognosis of colorectal cancer patients has been substantially improved, and the survival time has also been prolonged to a certain extent (Baidoun et al. 2021; Kanth and Inadomi 2021). However, at present, although the survival rate of patients with early colorectal cancer can reach more than 80%, the survival rate of patients with advanced colorectal cancer is still less than 20%, so the overall prognosis of the disease has not reached a satisfactory degree (Dariya et al. 2020; Kanth and Inadomi 2021). Therefore, it is of great clinical significance to deeply explore and understand the molecular mechanism of colorectal cancer occurrence and development, identify colorectal cancer-related genes and their functions, and excavate more molecular targets or markers that can be used for disease diagnosis and treatment, so as to enhance the clinical precise treatment of colorectal cancer patients to improve the prognosis and survival of patients (Harada and Morlote 2020; Li et al. 2021a; Xie et al. 2020).

It is well-known that glucose metabolism is a major source of cellular energy. However, unlike normal cells which carry out glucose metabolism mainly through oxidative phosphorylation pathways, tumor cells rely on the glycolytic pathway for providing energy even under aerobic conditions, a phenomenon also known as the Warburg effect (Hay 2016; Nenkov et al. 2021; Vaupel and Multhoff 2021). Through glycolysis, a less efficient but faster pathway to produce adenosine triphosphate (ATP) compared with oxidative phosphorylation, tumor cells acquire energy while producing a hypoxic and high lactate tumor microenvironment in the lesion area through the accumulation of metabolites. On the one hand, this promotes the rapid proliferation of tumor cells, on the other hand, it inhibits the immune monitoring functions of tumor-related immune cells so as to achieve immune escape (Pascale et al. 2020; Yuan et al. 2022). The main route that tumor cells enhance their own glycolysis is the overexpression of glycolysis-related enzymes as well as enhanced activity (Abbaszadeh et al. 2020; Park et al. 2020). The aldolase family contains three members, ALDOA, ALDOB, and ALDOC, which play important catalytic roles in glycolysis with glucose as substrate (Chang et al. 2018). Accordingly, numerous studies have shown that aldolase family members are upregulated in a variety of malignancies and are closely related to tumor progression (Bu et al. 2018; Huang et al. 2020; Li et al. 2021b; Niu et al. 2021). Notably, in addition to their own enzymatic functions, members of the aldolase family also could regulate tumor progression by regulating downstream signaling pathways such as Wnt/β-catenin through non-enzymatic functions (Caspi et al. 2014). Among the aldolase family, ALDOC is currently relatively poorly studied, and the aim of this study is to explore its function in colorectal cancer cell phenotype as well as glycolysis regulation.

Herein, the expression of ALDOC in colorectal cancer tissues and its correlation with tumor pathological features and patient survival were detected and analyzed, respectively. By constructing a colorectal cancer cell model that interferes with ALDOC expression, ALDOC-induced regulation of colorectal cancer cell phenotype as well as glycolysis was studied. More importantly, further studies showed that in addition to its own enzyme function, ALDOC may also be able to promote glycolysis of tumor cells and promote tumor progression at the in vitro as well as in vivo levels by promoting the transcriptional activation of another glycolysis-related enzyme PGK1 by HIF-1α.

## Materials and methods

### Clinical specimen collection and immunohistochemical staining

Tissue chips YBR-HCol119-M001-M015 and YBR-HCol119-M002-M021 were procured from Shanghai Yibeirui Biomedical Science and Technology Co., Ltd., encompassing 110 para-carcinoma tissues and 98 colorectal cancer tissues, respectively. The chips underwent antigen retrieval using citrate buffer and were subsequently treated with 3% H_2_O_2_ and 5% serum for blocking. Immunostaining was performed using an anti-ALDOC antibody (1:100, #14844-1-AP, Proteintech Group, Inc.) followed by HRP-conjugated goat anti-rabbit IgG (1:400, #ab97080, Abcam) incubation at 37 °C for 1 h. The chips were then stained with DAB for 5 min and counterstained with hematoxylin (#BA4041, Baso) for 10 s. Following washing, the chips were sealed with neutral gum, and staining intensity along with the proportion of positive cells were observed under a microscope. The product of the positive cell score and the staining intensity score served as the determination of IHC, where a higher score indicated elevated protein expression. The staining of the tissue chips was performed upon the approval of Tongji Hospital Ethics Committee.

### Bioinformatics analysis based on TCGA

Colorectal cancer and normal tissue samples transcriptomic datasets were obtained from The Cancer Genome Atlas (TCGA) *via* the Genomic Data Commons (GDC) portal (https://portal.gdc.cancer.gov/). RNA-sequencing (RNA-seq) data was processed and normalized using the DESeq2 package in R. Prognostic analysis was conducted on CRC samples from TCGA, accessed through cBioPortal (https://www.cbioportal.org/), focusing on the clinical relevance of ALDOC and PGK1. The correlation between gene expression levels was assessed using the cor.test function in R, employing Spearman’s rank correlation for monotonic relationships and Pearson’s correlation for linear dependencies.

### Cell culture

Normal human colorectal mucosal cells FHC and colorectal cancer cell lines SW480, RKO, HCT116, CACO2 and DLD-1 were purchased from the American Type Culture Collection (ATCC, Manassas, VA, USA) and all cells were incubated at 37 °C in a humidified atmosphere containing. FHC, HCT116 and RKO were grown in 90% RPMI-1640 with 10% fetal bovine serum (FBS, Invitrogen). SW480cells were grown in 90% IMDM with 10% fetal bovine serum (FBS, Invitrogen). CACO2 and DLD-1 cells were grown in Dulbecco’s modified Eagle’s medium (DMEM; Gibco, Gaithersburg) with 10% FBS.

### Construction of cell models

The interference target sequences of ALDOC were designed as follows: ALDOC-1, GACCTCAAACGTTGTCAGTAT; ALDOC-2, CAGAAAGATGATAATGGTGTT; ALDOC-3, TATTGTGGAACCTGAAATATT. The interference target sequences of PGK1 were as follows: PGK1-1, CCACAGAAGGCTGGTGGGTTT; PGK1-2, AATGTCCAAAGCTGAGAAGAA; PGK1-3, GCTGACAAGTTTGATGAGAAT. Then these sequences were synthesized as single stranded DNA oligo and annealed to form double-stranded DNA oligo. RNA interference recombinant plasmids were constructed by linking double-stranded DNA oligo to the linearized vector. Using ALDOC and HIF1A gene as template, primers were designed, and the target gene fragment was prepared by PCR amplification. The gene overexpression plasmids were obtained by recombination of the linearized vector and the target gene fragment in vitro. The recombinant plasmids were mixed with lentivirus packaging helper plasmids and then dropped into 293T cell medium for cultureing 6 h. The medium was replaced with medium containing 10% FBS, and the cells were continued to be cultured at 37℃ in air containing 5% CO_2_ for 72 h. Cell supernatants were collected to Lentiviruses containing target sequences were harvested. 2–3 × 10^8^ TU/mL lentivirus infection solution was added to the RKO and HCT116 cell medium (1.5 × 10^5^ cells). After 72 h, and the fluorescence was observed under a microscope.

### Real-time quantitative reverse transcription

Total RNA was extracted from cells lysed by Trizol (Sigma) and quantified using a Nanodrop 2000/2000 C spectrophotometer (Thermo). The RNA was reverse-transcribed into cDNA using Hiscript QRT supermix for qPCR (+ gDNA WIPER) (Vazyme). The reaction system was prepared with cDNA, forward and reverse primers, and other reagents in the appropriate proportions. Real-time PCR was performed using a Real Time PCR instrument (ABI) under the following conditions: initial denaturation at 95 °C for 30 s, followed by 40 cycles of denaturation at 95 °C for 10 s, annealing at 60 °C for 30 s, and extension at 72 °C for 30 s. A melting curve analysis was conducted to ensure the specificity of the PCR products. The 2^−ΔΔCt^ method was used to calculate the relative levels of gene expression. The primer sequences used are listed in Table S1.

### Western blot (WB)

The concentration of total Protein extracted from the cells was measured by BCA Protein Assay Kit (HyClone-Pierce). 20–30 µg protein was subjected to sodium dodecyl sulfate-polyacrylamide gel electrophoresis (SDS-PAGE) in 10–12% separation gel, and then transferred onto PVDF membranes. PVDF membranes were incubated with 1 × TBST containing 5% skim milk for blocking. Primary and secondary antibodies were used to treat PVDF membranes at 37 °C for 2 h successively. PVDF membranes were attained with immobilon Western chemiluminescent HRP Substrote kit (Millipore). Chemiluminescence was performed and imaged using Chemiluminescence imaging system (GE). The primary antibodies were collected in Table S2.

For Co-Immunoprecipitation, 1.0 mg protein was incubated with antibody overnight at 4 °C, and incubated with 20 µL beads for 2 h at 4 °C. Protein-antibody-beads complexes were reacted with anti-HIF-1α and anti-ALDOC antibodies, respectively, by WB. Chemiluminescence was performed and imaged using Chemiluminescence imaging system. The primary and secondary antibodies used in this assay was listed in the western blot section.

### Celigo cell counting assay

After 72 h of lentivirus infection, RKO and HCT116 cells were resuspended and transferred to 96-well plates with 3000 or 2500 cells per well. The same field in a 96-well plate was scanned with Celigo Image Cytometry (Nexcelom) at the same time point for 5 consecutive days, and cell counts were performed. The cell growth curve was plotted with the proliferation factor as the ordinate and the time point as the abscissa.

### Cell counting Kit-8 (CCK-8) assay

A total of 2000 lentivirus infected RKO and HCT116 cells were plated in 100 µL of complete culture medium per well, spanning across five 96-well plates. Commencing on the second day following seeding, and proceeding until 4 h prior to the predetermined conclusion of the experiment, 10 µL of CCK-8 reagent was introduced into each well and incubate at 37 °C for an extra 4 h. Absorbance at a wavelength of 450 nm was measured using a microplate reader (Tecan Infinite). The optical density (OD) values for all wells are documented, and subsequently, cell viability was evaluated.

### Cell apoptosis assay

RKO and HCT116 cells, after infection, were cultivated in a 6-well plate at a volume of 2 mL per well for 5 days until they reached a confluence of 85%. The cells were harvested, and then cell pellets were resuspended to achieve a concentration of 1 × 10^7^ cells/mL. 100 µL suspension was taken and mixed with 5 µL of annexin V-APC for staining 10 min. Subsequently, the mixture underwent centrifugation at 1500 rpm for 3 min, followed by resuspending the cell pellet in 100 µL of 1 × binding buffer. Next, 5 µL of PI was introduced for dual staining, then transferred into tubes for flow cytometry (Millipore). The number of cells undergoing apoptosis was quantified.

### Cell cycle assay

When lentivirus infected RKO and HCT116 cells in the 6-cm dishes from the experimental group had grown to reach approximately 70% confluence, they were harvested. Subsequently, a centrifugation process at 1200 rpm for 5 min ensued and the cell pellets were then washed with pre-cooled PBS at 4 °C. Following, the cells were fixed in pre-cooled 70% ethanol for 1 h at 4 °C, and centrifugated at 1500 rpm for 5 min to remove the ethanol. After washing with PBS, the cell pellets were resuspended in 1 mL of cell staining solution (40 × PI, 2 mg/mL: 100 × RNase, 10 mg/mL: 1 × PBS = 25: 10: 1000). Ultimately, the prepared samples were subjected to flow cytometry (Guava easyCyte HT, Millipore) for the purpose of quantifying and analyzing the population of cells in the G1, S, and G2 phase.

### Wound healing assay

A monolayer of 5 × 10^4^ RKO and HCT116 cells per well was established within a culture system consisting of 100 µL of medium per well in a 96-well plate. The formation of a wound in the cell monolayer was facilitated by using a 96 Wounding Replicator. Images capturing the immediate state of the wounds (0 h) were taken. Thereafter, appropriate time points (24–48 h) were selected and the wounded areas are imaged again using Cellomics for further documentation and cellular migration assessing.

### Transwell assay

A quantity of 5 × 10^4^ lentivirus infected RKO and HCT116 cells per well was seeded in a 24-well plate, with an inner chamber volume set at 100 µL per well and an outer chamber filled with 600 µL per well. Cells were then subjected to incubation within the Transwell for a duration of 72 h, allowing for migration or invasion. 400 µL of staining solution was introduced into each insert for a period of 5 min, coloring the cells which have migrated or invaded through the membrane to its underside. Images were captured under a microscope to visually document and assess the capability of cells to migrate or invade through the pores of the Transwell membrane.

### Dual-Luciferase reporter assay

PCR was utilized to amplify the full length of PGK1 and wild-type cDNA fragments, which possessed a predicted miRNA binding site of PGK1’s 3’-UTR. Subsequently, mutated fragments were generated through the use of overlap extension PCR. Afterward, both the wild-type PGK1 and the 3’UTR region of PGK1, and their respective mutant sequences were individually recombined into GL002 pGL-mcs-Luciferase-SV40poly(A) vectors (Promega, USA). HEK293T cells were then co-transfected with WT or MUT vectors, along with HIF1A and ALDOC or their respective control mimics, utilizing 0.8 mg/mL lipo-3000 (Merck, USA). Following a 48 h incubation period, the luciferase activity was detected using a Promega Dual-Luciferase system (Promega, USA), and the activity of firefly luciferase was normalized with Renilla luciferase activity.

### Chromatin Immunoprecipitation assay (ChIP)

The ChIP assay was performed using SimpleChIP^®^ Enzymatic Chromatin IP Kit (CST, USA) according to the operating manual. Initially, 1% formaldehyde was used to crosslink the protein and DNA. Following cell lysis, chromatin was fragmented into minute pieces via 0.5 µL micrococcal nuclease. 5 µg anti-HIF1A antibody or normal rabbit IgG control antibody was utilized to immunoprecipitated the DNA-protein complexes. The DNA-protein crosslinking was reversed through the application of 6 µL of NaCl and 2 µL of proteinase K treatment. This step preceded DNA purification and recovery. Ultimately, the retrieved DNA was subjected to RT-qPCR for further analysis. The primer sequences used for the ChIP assay are listed as follows.

PGK1 Forward 5’-GGCTGAGGCAGGAGAATCAC-3’.

PGK1 Reverse 5’-GTCTCACTCTGTCGCCCAAG-3’.

### Nucleus–cytoplasmic fractionation assay

For total protein extraction, cells were lysed using IP lysis buffer, centrifuged at 12,000 rpm for 10 min, and the supernatant was transferred to a centrifuge tube. Loading buffer was added, and the mixture was centrifuged again at 12,000 rpm for 1 min. The samples were then analyzed using Western blotting (WB). Proteins were separated on an SDS-PAGE gel and transferred to PVDF membranes. The PVDF membranes were blocked with 5% non-fat milk in TBST solution for 1 h. The membranes were then incubated overnight at 4 °C with primary antibodies (Table S2). After washing the PVDF membranes three times with TBST, the membranes were incubated with the secondary antibody (Table S2) for 1 h at room temperature. Following three additional washes with TBST, the membranes were detected using the Millipore immobilon Western Chemiluminescent HRP Substrate kit (Millipore, #RPN2232, USA).

### Xenograft tumor model

BALB/c male nude mice (4 weeks old) were purchased from GemPharmatech Co., Ltd (Jiangsu, China). A total of 10 mice were used in this study and randomly divided into two groups (*n* = 5 per group) to ensure balanced experimental conditions. All mice were housed in specific pathogen-free (SPF) environments under a 12 h light/dark cycle at a temperature of 18–25 °C and humidity of 50–60%. RKO (1 × 10^7^) cells were injected into the flank of the mice. A caliper was used to measure tumor volumes. The mice were sacrificed after 27 days, and tumor volumes and weight were measured. The length and width of the tumors were measured to calculate the tumor volume using the formula (Tumor volume = π/6 × L × W^2^). Additionally, Ki67 staining with anti-Ki67 (1:50, #ab16667, Abcam, USA) and HRP-conjugated goat anti-rabbit IgG (1:400, #ab97080, Abcam, USA) was performed to detect the ALDOC expression in tumor tissues. All animal procedures were conducted in accordance with Tongji Hospital Ethics Committee.

### ATP, glucose and lactate determination

ATP, glucose and lactate content were determined using Solarbio ATP Content detection reagent kit (Beijing, China), the Solarbio Glucose content detection kit (Beijing, China), and the Lactic acid content detection reagent kit (Beijing, China) respectively. For ATP detection, RKO or HCT116 cell samples were prepared. The 0.625 µmol/mL ATP standard solution was added to the cell samples. The optical density (OD) value at 340 nm was measured. Finally, ATP content was calculated. For glucose detection, RKO or HCT116 cell samples were prepared. Next, the indicated solution and mixed reagents were added into cell samples. The OD value was measured at 505 nm, and the glucose content was calculated. For lactate detection, after preparing RKO or HCT116 cell samples, the indicated reagent was added to cell samples. Finally, the OD value measured at 570 nm, as well as lactate content, was calculated.

### Extracellular acidification rates (ECAR), oxygen consumption rates (OCR) measurement

Extracellular acidification rates (ECAR) were measured using Extracellular acidification Rate Assay Kit (BestBio, Shanghai, China) followed by manufactory’s instruction. In brief, RKO or HCT116 cells were plated in 96 well with 2.5 × 10^3^ cells/well separately and cultured at 37 °C in a humidified incubator containing 5% CO_2_. Then, 10 µL BBcellProbe^®^ P61 acidic fluorescent probe were added to each well. The fluorescence intensity was measured using microplate reader at an excitation wavelength of 488 nm and an emission wavelength of 580 nm. Oxygen consumption rates (OCR) were measured using Oxygen Consumption Assay Kit (BestBio, Shanghai, China) according to manufactory’s instruction. Briefly, RKO or HCT116 cells were plated in 96 well with 2.5 × 10^3^ cells/well separately and cultured at 37 °C in a humidified incubator containing 5% CO_2_. After 4 µL BBoxiProbe^®^ R01 oxygen fluorescence probe was added, the 100 µL blocking liquid were added to each well. The fluorescence intensity was measured using microplate reader at an excitation wavelength of 455 nm and an emission wavelength of 603 nm. The ECAR and OCR curves were plotted separately.

### Statistical analysis

The data were presented as the mean ± Standard Deviation (SD) at least three independent determinations. The correlation between ALDOC expression and the clinicopathological features of the patients was performed with the Chi-square test or Mann Whitney U analysis, followed by Spearman’s correlation analysis. Multiple groups were analyzed using ANOVA. The Kaplan-Meier method was employed to analyze the overall survival (OS) curve, which was further compared using a log-rank test. The quantitative data were compared using Mann-Whitney U test or Student’s t-test. A value of *P* < 0.05 were considered statistically significant.

## Results

### ALDOC is upregulated in CRC tissues and predicts poor prognosis

**Fig. 1:**
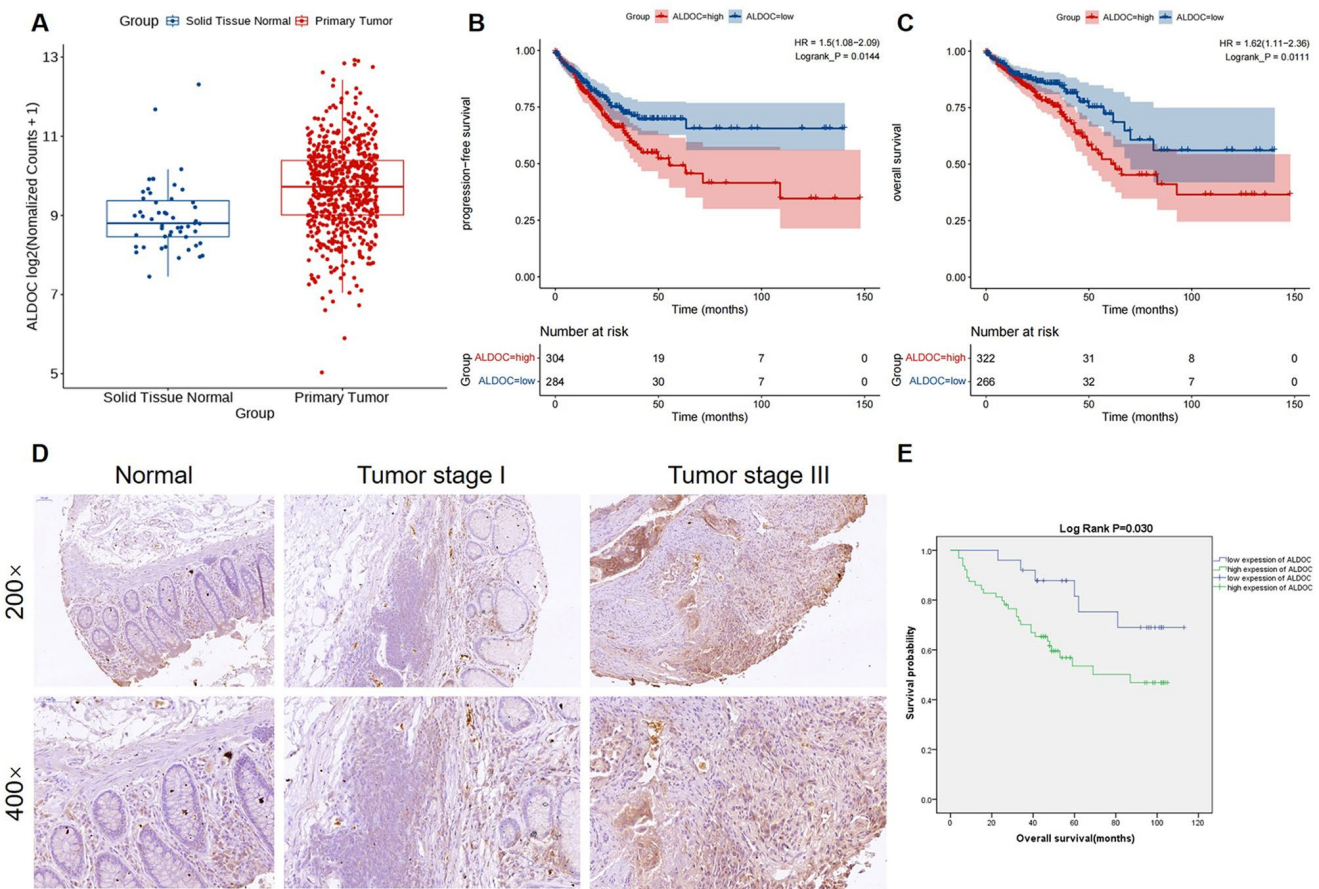
ALDOC is upregulated in CRC tissues and predicts poor prognosis. (**A**) The differential expression of ALDOC in CRC tissues and normal tissues was analyzed based on TCGA-COAD + READ datasets. (**B**, **C**) The progression-free survival (**B**) and overall survival (**C**) were analyzed based on TCGA database. (**D**) A tissue microarray containing CRC tissues and normal tissues were subjected to immunohistochemical staining for detecting expression of ALDOC. Representative images were shown. (**E**) Overall survival was analyzed based on the results of immunohistochemical staining

In our investigation of ALDOC expression patterns in CRC, we initially analyzed transcriptomic expression profiles from TCGA COAD and READ datasets. Our analysis revealed a significant upregulation of ALDOC expression in tumor tissues compared to normal tissues (Fig. 1A). Using median for dividing high/low ALDOC expression groups, survival analysis further demonstrated that patients with high ALDOC expression in tumor tissues exhibited relatively shorter progression-free survival and overall survival (Fig. 1B and C). These findings were validated in locally collected patient-derived tissue samples, where immunohistochemical (IHC) staining (Fig. 1D) and statistical analysis (Table 1) indicated significantly elevated ALDOC expression in tumor tissues compared to adjacent normal tissues. Moreover, it was also demonstrated that ALDOC expression increases significantly along with the elevation of the malignancy of tumor, such as more advanced T stage, N stage, pathological stage (Fig. 1D), and more serious lymph node invasion (Table 2 and S3). Survival analysis based on IHC staining also suggested that high ALDOC expression (also divided by IHC score median) predicted poorer prognosis (Fig. 1E). Overall, our results highlight the upregulation of ALDOC in CRC, its association with adverse prognosis, and underscore the need for further investigation into its specific functions.

**Table 1:** Expression patterns of ALDOC in colorectal cancer tissues and normal tissues revealed in immunohistochemistry analysis

ALDOC expression	Tumor tissue		Normal tissue	
	Cases	Percentage	Cases	Percentage
Low	29	29.6%	110	100%
High	69	70.4%	0	0%

*P* < 0.001

**Table 2:** Relationship between ALDOC expression and tumor characteristics in patients with colorectal cancer

Features	No. of patients	ALDOC expression		*P* value
		low	high	
All patients	98	29	69	
Age (years)				0.726
≤ 62	48	15	33	
> 62	50	14	36	
Gender				0.953
Male	57	17	40	
Female	41	12	29	
Tumor size				0.699
≤ 4	57	16	41	
> 4	41	13	28	
Differentiation				0.102
low	8	1	7	
medium	87	26	61	
high	3	2	1	
Stage				< 0.001
I	9	8	2	
II	37	14	23	
III	44	7	37	
IV	8	0	8	
T Infiltrate				0.004
T1	2	2	0	
T2	13	8	5	
T3	46	12	34	
T4	37	7	30	
lymphatic metastasis (N)				0.001
N0	49	22	27	
N1	32	5	27	
N2	17	2	15	
Metastasis				0.057
M0	90	29	61	
M1	8	0	8	
Lymph node invasion				0.001
NO	49	22	27	
YES	49	7	42	
Vascular invasion				0.631
NO	93	28	65	
YES	5	1	4	

### ALDOC could regulate CRC cell phenotypes in vitro

**Fig. 2:**
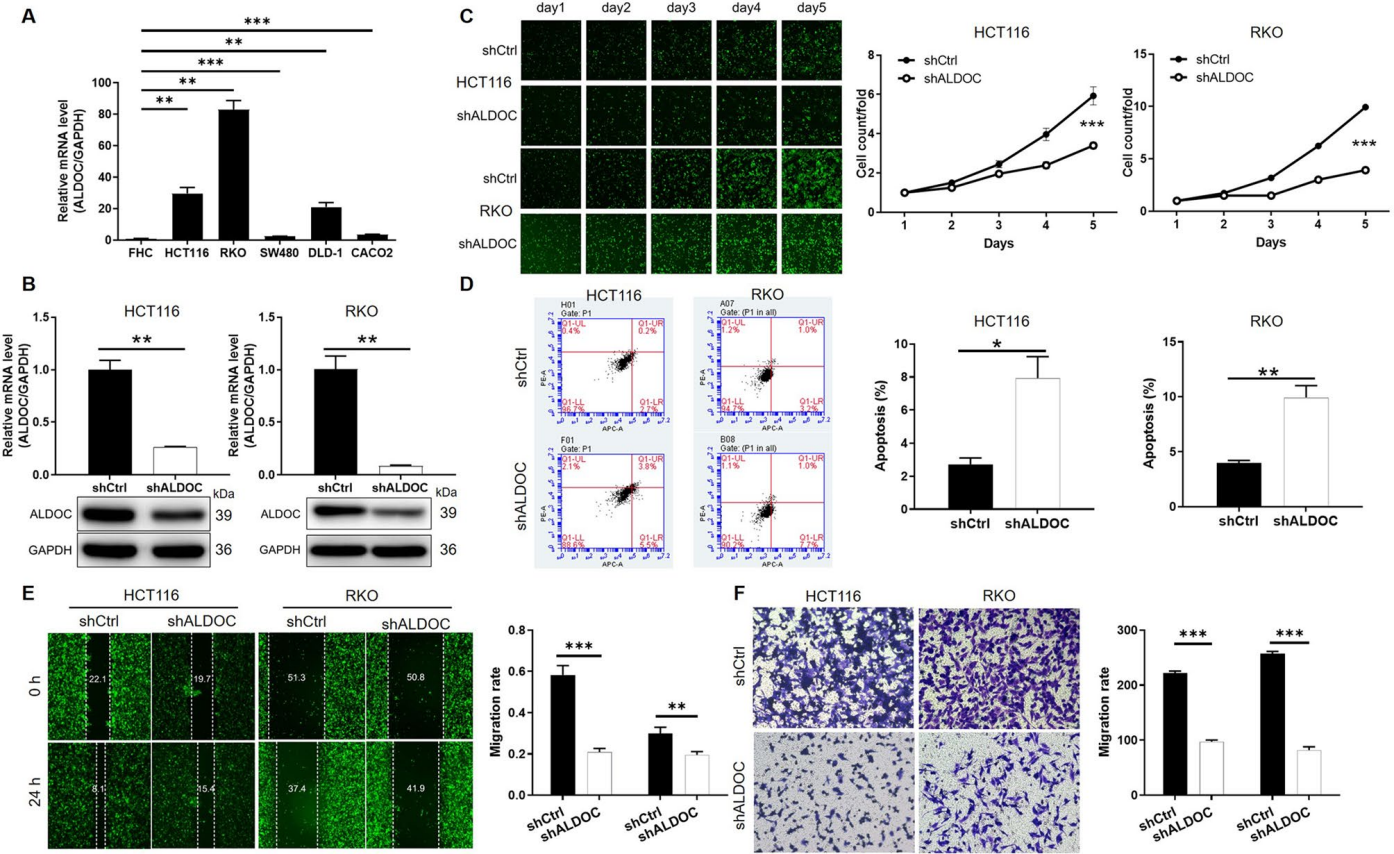
ALDOC could regulate CRC cell phenotypes in vitro. (**A**) The endogenous expression of ALDOC was detected by qPCR in human normal intestinal epithelial cell line FHC and several CRC cell lines including HCT116, RKO, SW480, DLD-1, and CACO2. (**B**) The knockdown efficiencies of ALDOC in HCT116 and RKO cells were evaluated based on mRNA and protein levels. (**C**) Celigo cell-counting assay was performed to assess the effects of ALDOC knockdown on cell proliferation of HCT116 and RKO cells. (**D**) Flow cytometry was performed in shCtrl and shALDOC cells for examining cell apoptosis. (**E**, **F**) Cell migration of HCT116 and RKO cells with or without ALDOC knockdown was evaluated by wound-healing assay (**E**) and transwell assay (**F**). Data were drawn as mean ± SD (*n* ≥ 3). * *P* < 0.05, ** *P* < 0.01, *** *P* < 0.001

Prior to conducting in vitro experiments, we assessed the endogenous mRNA expression levels of ALDOC in human normal intestinal epithelial cell line FHC and several CRC cell lines including HCT116, RKO, SW480, DLD-1, and CACO2. Our findings revealed that ALDOC expression was generally higher in CRC cell lines compared to normal cells (Fig. 2A). Subsequently, we selected HCT116 and RKO cells with relatively high ALDOC expression, along with the most efficient of the three shRNAs designed for ALDOC knockdown (Figure S1), to establish ALDOC knockdown CRC cell models. The knockdown efficiency was validated using qPCR and WB (Fig. 2B). Assessment of cell proliferation using Celigo assays demonstrated that ALDOC knockdown significantly inhibited cell proliferation rates (Fig. 2C). Additionally, flow cytometry analysis revealed that ALDOC knockdown increased the proportion of apoptotic cells (Fig. 2D), reduced the proportion of cells in the S phase, and induced G2 phase cell cycle arrest (Figure S2). Furthermore, wound-healing assay (Fig. 2E) and Transwell assay (Fig. 2F) consistently showed that ALDOC knockdown suppressed the migration ability of CRC cells. Collectively, the results of our in vitro experiments indicate that ALDOC significantly regulates the phenotypes of CRC cells, particularly affecting cell proliferation, apoptosis, cell cycle progression, and migration ability.

### ALDOC knockdown suppress CRC tumor growth in vivo

**Fig. 3:**
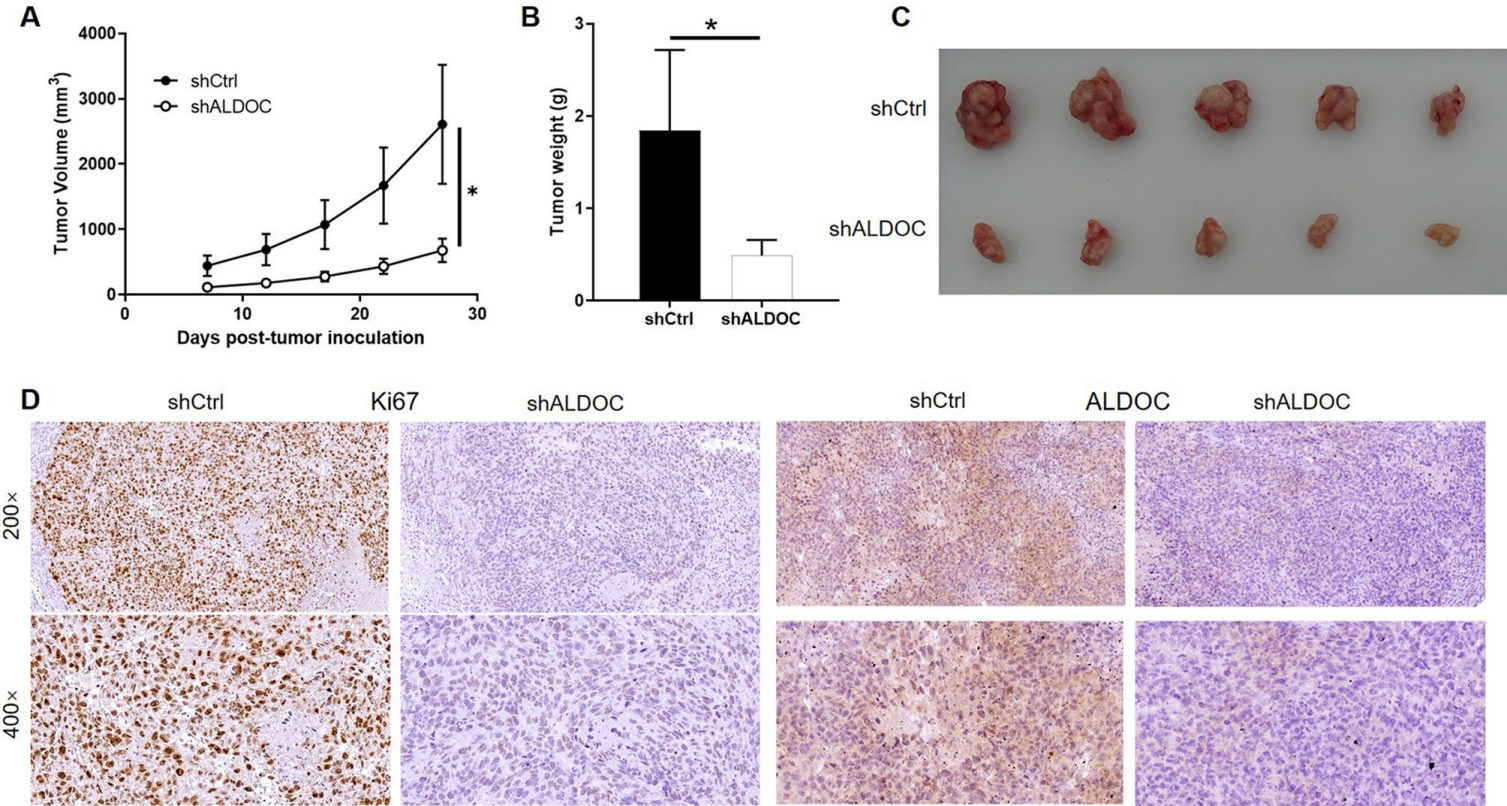
ALDOC knockdown suppress CRC tumor growth in vivo. (**A**) Xenograft mice models was constructed based on RKO cells with or without ALDOC knockdown. The volume of the xenografts was recorded during animal culturing. (**B**, **C**) After sacrificing the mice models, xenografts were collected for weighting (**B**) and taking photos (**C**). (**D**) Immunohistochemical staining was performed in xenografts to detect the protein level of Ki67 and ALDOC. Data were drawn as mean ± SD (*n* = 5). * *P* < 0.05

In order to investigate the in vivo effects of ALDOC knockdown on CRC tumor growth, we established an animal model by injecting RKO cells with or without ALDOC knockdown into the right flank of nude mice. Subsequent observation of subcutaneous tumor growth revealed a significant deceleration in tumor growth rate in the shALDOC group compared to the shCtrl group (Fig. 3A), resulting in smaller and lighter tumors in the former (Fig. 3B and C). Immunohistochemical (IHC) analysis of the tumor tissues further confirmed the downregulation of Ki67 expression, a marker of cell proliferation, in tumors derived from the shALDOC group, validating the efficacy of ALDOC knockdown in the tumor tissues (Fig. 3D). Collectively, these findings demonstrate that ALDOC knockdown attenuates tumor growth in vivo, suggesting a potential therapeutic strategy for colorectal cancer.

### ALDOC May promote PGK1 expression through interacting HIF1A and activating PGK1 transcription

**Fig. 4:**
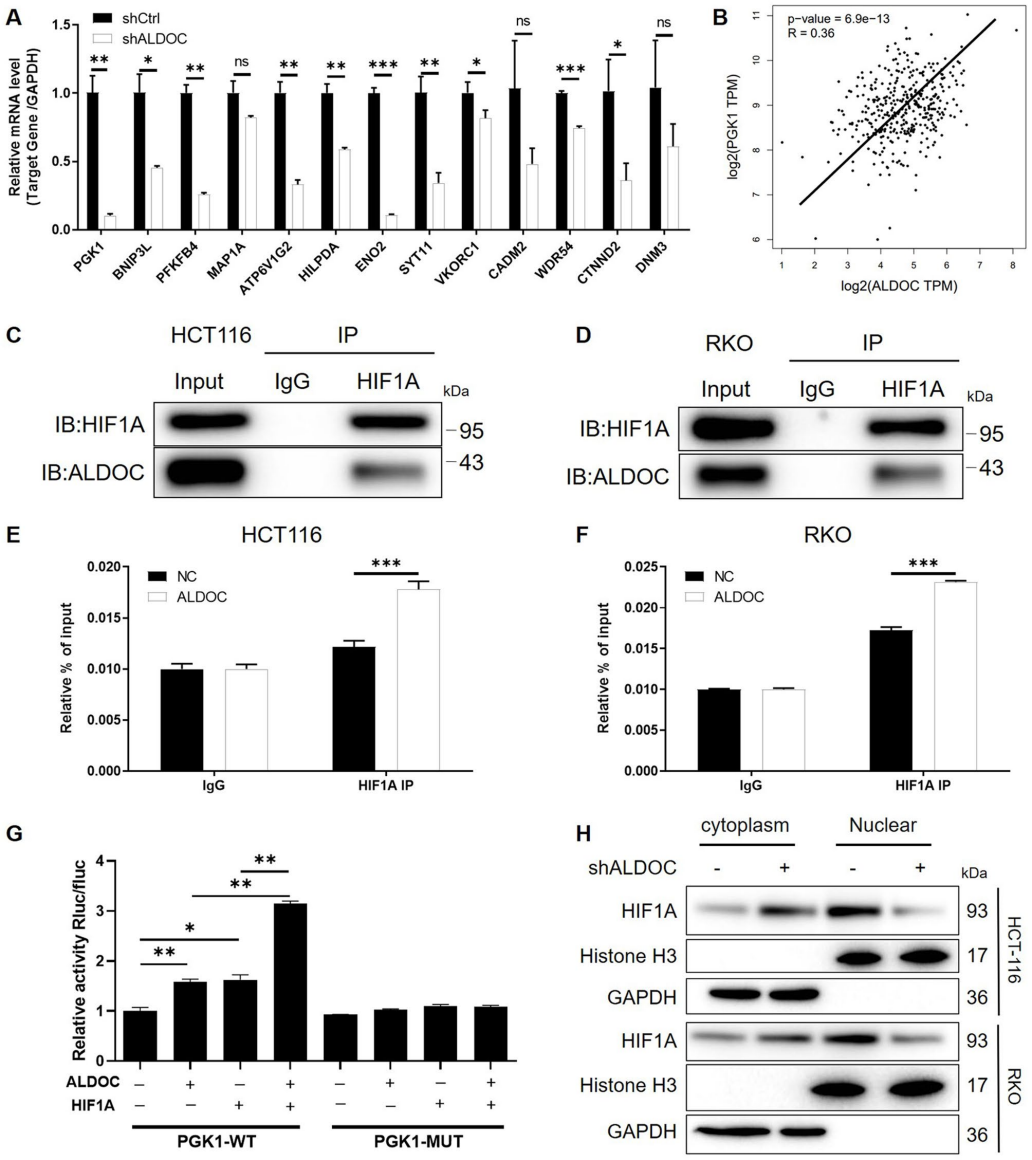
ALDOC may promote PGK1 expression through interacting HIF1A and activating PGK1 transcription. (**A**) The expression of several co-expressed genes of ALDOC predicted by Coexpedia database in CRC cells with or without ALDOC knockdown was detected by qPCR. (**B**) The correlation between ALDOC and PGK1 in CRC was analyzed based on TCGA-COAD + READ datasets. (**C**) Immunoprecipitation was performed by anti-HIF1A in HCT116 cells followed by the detection of HIF1A and ALDOC by WB. (**D**) Immunoprecipitation was performed by anti-HIF1A in RKO cells followed by the detection of HIF1A and ALDOC by WB. (**E**, **F**) Chromatin immunoprecipitation was used to detect the enrichment of PGK1 promoter in complex precipitated by anti-HIF1A. (**G**) PGK1 promoter plasmid was transfected into HEK293T cells with/without ALDOC or HIF1A overexpression. Cells were harvested after 24 h transfected and promoter activity was measured by Dual-Luciferase Reporter assay system. (**H**) WB analysis of HIF1A in cytoplasmic and nucleus fractions of HCT116 and RKO cells. Histone H3 and GAPDH were used as nucleus and cytoplasmic markers, respectively. Data were drawn as mean ± SD (*n* ≥ 3). * *P* < 0.05, ** *P* < 0.01, *** *P* < 0.001

To further explore the downstream pathways regulated by ALDOC in CRC, we conducted an analysis of ALDOC’s co-expressed genes using the Coexpedia online tool (Yang et al. 2017), followed by experimental validation *via* qPCR (Fig. 4A). Given the known role of ALDOC in glycolysis regulation, particular attention was drawn to PGK1, a gene associated with glycolysis (Liu et al. 2024; Quan et al. 2023), among its co-expressed genes. Correlation analysis based on TCGA database (COAD + READ dataset) confirmed a positive correlation between ALDOC and PGK1 expression in CRC tissues (Fig. 4B). It was also discovered that endogenous mRNA levels of PGK1 in CRC cell lines are generally higher than FHC cell line (Figure S3). Additionally, TRRUST database revealed HIF1A as one of the known transcriptional activators of PGK1, while STRING database indicated a potential protein-protein interaction between ALDOC and HIF1A, which was also confirmed by co-immunoprecipitation experiments (Fig. 4C and D). This led us to hypothesize that ALDOC might regulate HIF1A-mediated PGK1 transcription through interaction with HIF1A. Chromatin immunoprecipitation assays further demonstrated significant enrichment of PGK1 promoter in the protein-DNA complexes immunoprecipitated with HIF1A antibody, particularly enhanced in ALDOC-overexpressing conditions (Figure S4 and 4E-4F). This suggests capability of HIF1A to bind to the PGK1 promoter and initiate its transcription, which is augmented by ALDOC. Dual-luciferase reporter assays revealed that both HIF1A and ALDOC overexpression enhanced the activity of luciferase vector loading PGK1 promoter, synergistically, with no effect observed on the mutated binding site of the PGK1 promoter (Fig. 4G). Finally, nuclear-cytoplasmic fractionation followed by WB analysis showed a significant decrease in nuclear expression of HIF1A upon ALDOC knockdown, weakening its transcriptional regulatory effect on PGK1 (Fig. 4H). These results collectively suggest a regulatory role of ALDOC in modulating HIF1A-mediated PGK1 transcriptional activation, shedding light on a novel regulatory axis in CRC glycolysis regulation.

### ALDOC/HIF1A/PGK1 axis corporately regulates CRC cell phenotypes

**Fig. 5:**
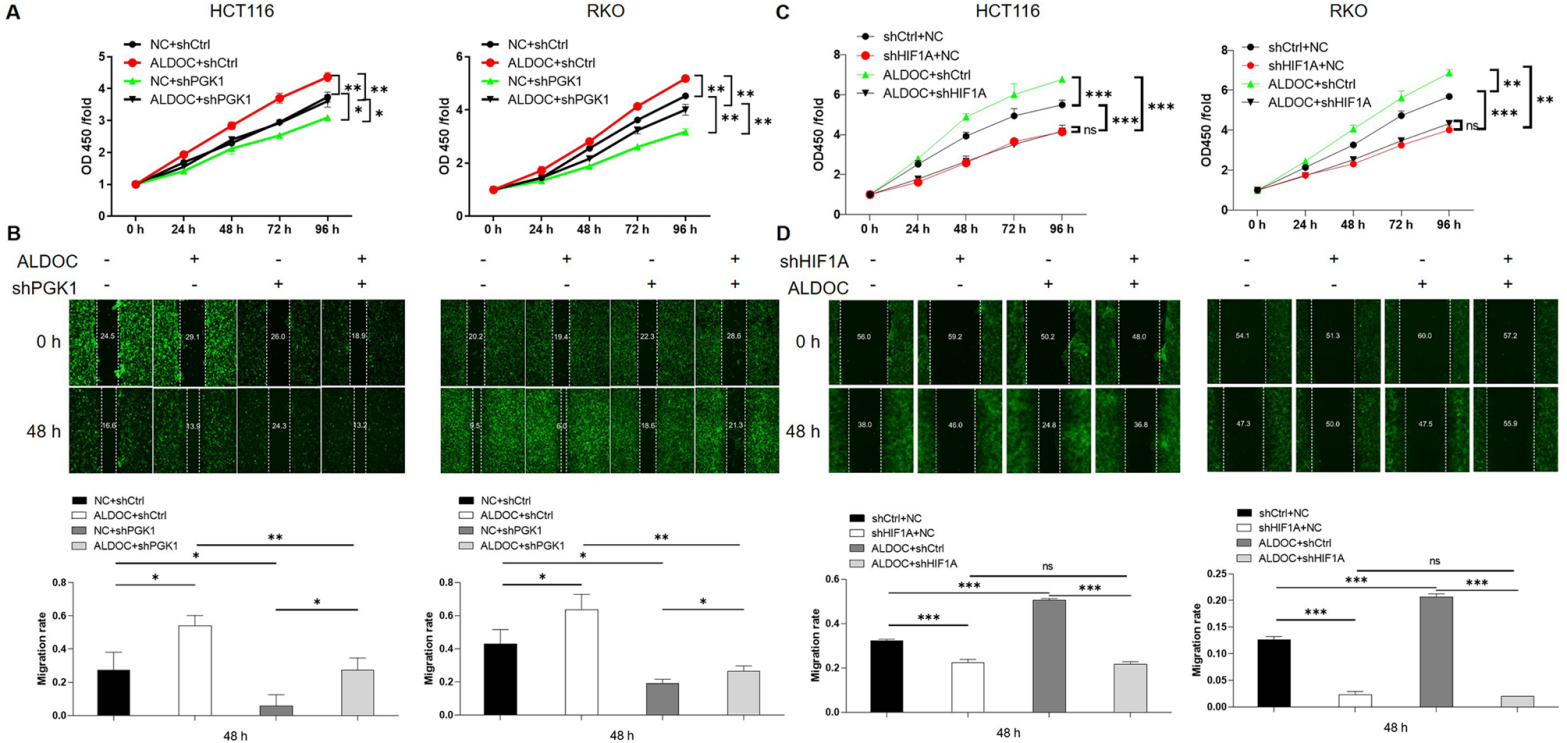
ALDOC/PGK1 axis corporately regulates CRC cell phenotypes. (**A**) Proliferation of HCT116 and RKO cells with ALDOC overexpression, PGK1 knockdown or simultaneous ALDOC overexpression and PGK1 knockdown was tested by CCK8 assay. (**B**) Migration of HCT116 and RKO cells with ALDOC overexpression, PGK1 knockdown or simultaneous ALDOC overexpression and PGK1 knockdown was tested by wound-healing assay. (**C**) Proliferation of HCT116 and RKO cells with ALDOC overexpression, HIF1A knockdown or simultaneous ALDOC overexpression and HIF1A knockdown was tested by CCK8 assay. (**D**) Migration of HCT116 and RKO cells with ALDOC overexpression, HIF1A knockdown or simultaneous ALDOC overexpression and HIF1A knockdown was tested by wound-healing assay. Data were drawn as mean ± SD (*n* ≥ 3). * *P* < 0.05, ** *P* < 0.01

Subsequently, we conducted in vitro functional rescue experiments to explore the synergistic regulatory effects of ALDOC and PGK1 on CRC cell phenotypes. Utilizing lentiviral vectors validated for effective ALDOC overexpression and PGK1 knockdown via qPCR and WB (Figure S4-S5), we established CRC cell models with ALDOC overexpression, PGK1 knockdown, and simultaneous ALDOC overexpression coupled with PGK1 knockdown. Consistent with previous findings, ALDOC overexpression and PGK1 knockdown individually exhibited significant promotion and inhibition of CRC cell proliferation and migration, respectively (Fig. 5A and B). More importantly, when PGK1 expression was attenuated in ALDOC-overexpressing cells, the enhanced proliferation and migration induced by ALDOC overexpression were partially attenuated (Fig. 5A and B). Similarly, knockdown of HIF1A could also attenuate the enhancement of cell proliferation and cell migration induced by ALDOC overexpression (Fig. 5C and D). These results indicate a certain dependence of ALDOC-related regulatory effect on CRC cell phenotype on PGK1, suggesting a potential synergistic interplay between ALDOC and PGK1 in CRC pathogenesis.

### ALDOC May promote aerobic Glycolysis of CRC through regulating PGK1

**Fig. 6:**
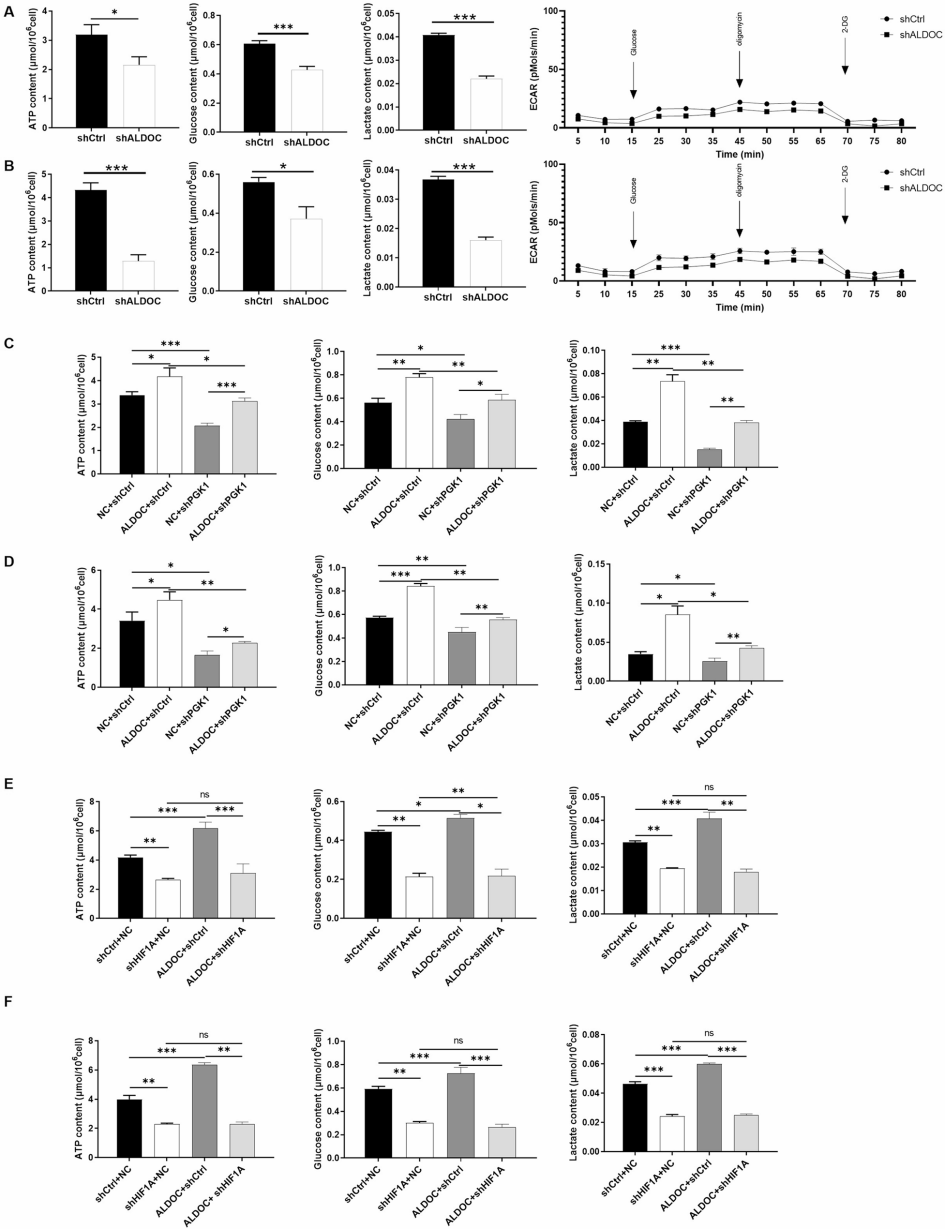
ALDOC may promote aerobic glycolysis of CRC through regulating PGK1. (**A**) ATP content, glucose content, lactate content and ECAR were evaluated in HCT116 cells with or without ALDOC knockdown. (**B**) ATP content, glucose content, lactate content and ECAR were evaluated in RKO cells with or without ALDOC knockdown. (**C**) ATP content, glucose content and lactate content were detected in HCT116 cells with ALDOC overexpression, PGK1 knockdown or simultaneous ALDOC overexpression and PGK1 knockdown. (**D**) ATP content, glucose content and lactate content were detected in RKO cells with ALDOC overexpression, PGK1 knockdown or simultaneous ALDOC overexpression and PGK1 knockdown. (**E**) ATP content, glucose content and lactate content were detected in HCT116 cells with ALDOC overexpression, HIF1A knockdown or simultaneous ALDOC overexpression and HIF1A knockdown. (**F**) ATP content, glucose content and lactate content were detected in RKO cells with ALDOC overexpression, HIF1A knockdown or simultaneous ALDOC overexpression and HIF1A knockdown. Data were drawn as mean ± SD (*n* ≥ 3). * *P* < 0.05, ** *P* < 0.01, *** *P* < 0.001

As previously discussed, both ALDOC and PGK1 are glycolysis-related proteins known to significantly regulate aerobic glycolysis in malignant tumors. Therefore, we proceeded to assess aerobic glycolysis-related indicators in CRC cells. As illustrated in Fig. 6A and B and S6, CRC cells with ALDOC knockdown exhibited significantly lower levels of ATP content, glucose uptake, and lactate production compared to shCtrl cells, alongside a decrease in extracellular acidification rate (ECAR) and an increase in oxygen consumption rate (OCR), indicating a notable regulatory effect of ALDOC on aerobic glycolysis in CRC cells. The results in Fig. 6C and D further demonstrate that while ALDOC overexpression and PGK1 knockdown individually modulate glycolysis indicators in CRC cells, notably, PGK1 knockdown partially reverses the promoting effect induced by ALDOC overexpression. Moreover, as a typical marker of aerobic glycolysis, HIF1A knockdown was also found to partially reverse the enhancement of glycolysis caused by ALDOC overexpression (Fig. 6E and F). This suggests an association between ALDOC-related regulation of aerobic glycolysis in CRC cells and PGK1.

## Discussion

Glucose metabolism reprogramming, AKA aerobic glycolysis, has been recognized as one of the hallmarks of malignant tumors (Hanahan 2022). Through glycolysis, tumor cells produce enough energy to supply the proliferation while creating a microenvironment conducive to their growth. In recent years, substantial evidence suggests that enhanced aerobic glycolysis plays an important role in the malignant progression of colorectal cancer and could be considered as a therapeutic target for the treatment of colorectal cancer (Nenkov et al. 2021). For example, it was recently reported that OTUB2 could boost the activity of PKM2, a key enzyme for glycolysis, through protein deubiquitination, thus promoting glycolysis of colorectal cancer and tumor progression (Yu et al. 2022). On the other hand, aldolases, including ALDOA, ALDOB and ALDOC, have been revealed to play a key role in glycolysis through catalyzing the metabolism of fructose-1, 6-diphosphate and fructose-1-phosphate. Reasonably, upregulation of aldolases has significant promotion effect on a variety of human cancers through enhancing glycolysis (Chang et al. 2018). For instance, high expression of ALDOA has been demonstrated to enhance aerobic glycolysis in intrahepatic cholangiocarcinoma and thereby promote proliferation and invasion of cholangiocarcinoma cells (Li et al. 2021b). Fan et al. showed that MUC16c promotes glycolysis and tumor progression of gallbladder carcinoma by binding to ALDOC and increasing its stability to regulate its glucose sensing ability mediated by the AMPK pathway (Zhang et al. 2017). Moreover, simple ALDOC knockdown is able to significantly inhibit glucose uptake and glycolysis of gallbladder cancer cells (Fan et al. 2020). Similarly, ALDOC also mediate the glycolysis-related tumor progression of breast cancer and negatively coordinates with poor prognosis of patients (Reinsborough et al. 2021). Importantly, in an association study of BRD9 with colon cancer conducted by Zhu et al., they found that the promoting effect of BRD9 on glucose metabolism reprogramming and tumor progression of colon cancer was partially derived from the enhanced acetylation modification of H3K27 in the ALDOC promoter region and the resulting transcriptional activation of ALDOC (Zhu et al. 2023). Our work, on the other hand, more clearly showed that ALDOC has a significant regulatory effect on glucose uptake, lactate production, and ATP production of colorectal cancer cells, indicative of its direct regulatory effect on colorectal cancer glycolysis. Accordingly, ALDOC also showed a significant promoting effect on the proliferation and migration phenotypes of colorectal cancer cells in vitro, and on tumor growth in vivo. All these evidences showed that ALDOC could promote glycolysis thereby the development and progression of colorectal cancer.

Through the study of aldolase function, it is gradually found that in addition to their own enzyme-related function in glycolysis, they can also play a role in the regulation of cellular function unrelated to their enzyme activity through the interaction with other molecules. For example, Wnt signaling is an important pathway that mediates the regulation of cancer cell phenotypes by the aldolase family independently of glycolysis (Caspi et al. 2014). Notably, in this respect, most studies concentrate on ALDOA and ALDOB, and the non-enzyme functions of ALDOC was rarely investigated and reported. Interestingly, during our exploration of downstream of ALDOC, its co-expression feather with PGK1, another key enzyme in glycolysis as well as in cancer development (Fu and Yu 2020; Nie et al. 2020), brings our eyes back to the relevant field of glycolysis. As a novel mechanism, we proposed that ALDOC may also be able to promote glycolysis through its promotion effects on the expression of PGK1 instead of its own glycolysis-related function. On the other hand, a large number of studies have shown that HIF-1α can activate the transcription of a variety of target genes and regulate the expression of key enzymes of glycolysis, thereby increasing glucose transport, increasing glycolytic energy supply, and promoting angiogenesis, and then enable tumors to keep developing under hypoxic conditions (Cheng et al. 2014; Kierans and Taylor 2021). PGK1 is one of the key enzymes of glycolysis that have long been known to be transcriptionally regulated by HIF-1α (Semenza 2010; Semenza et al. 1994). In this study, we showed that ALDOC interacts on protein level with HIF-1α and promotes binding of HIF-1α to the PGK1 promoter region, which may be an important mechanism by which ALDOC induces upregulation of PGK1 expression. Unsurprisingly, PGK1 also functions in concert with ALDOC to regulate glycolysis-related parameters as well as malignant phenotypes of colorectal cancer cells.

In conclusion, this study identified ALDOC/PGK1 axis as a key promotor of colorectal cancer development through inducing glycolysis. Importantly, in addition to acting as a glycolysis-related enzyme to induce glycolysis, ALDOC is also able to promote glycolysis by promoting HIF-1α-mediated transcriptional activation of PGK1. In summary, ALDOC may be a key molecular target to block glycolysis as well as tumor progression of colorectal cancer.

## Electronic supplementary material

Below is the link to the electronic supplementary material.

Supplementary Figure 1: The knockdown efficiencies of 3 shRNAs prepared for silencing ALDOC were assessed by qPCR in RKO cells. Data were shown as mean with standard deviation. Data were drawn as mean α SD (*n* ≥ 3). ** *P* < 0.01

Supplementary Figure 2: Flow cytometry was performed in shCtrl and shALDOC cells for examining cell cycle. Data were drawn as mean α SD (*n* ≥  3). ** *P* < 0.01, *** *P* < 0.001

Supplementary Figure 3: The endogenous expression of PGK1 was detected by qPCR in human normal intestinal epithelial cell line FHC and several CRC cell lines including HCT116, RKO, SW480, DLD-1, and CACO2. Data were drawn as mean α SD (*n* ≥ 3). ** *P* < 0.01, *** *P* < 0.001

Supplementary Figure 4: The overexpression efficiencies of ALDOC in HCT116 and RKO cells were evaluated based on mRNA and protein levels. Data were drawn as mean α SD (*n* ≥ 3). *** *P* < 0.001

Supplementary Figure 5: The knockdown efficiencies of PGK1 in HCT116 and RKO cells were evaluated based on mRNA and protein levels. Data were drawn as mean α SD (*n* ≥ 3). ** *P* < 0.01, *** *P* < 0.001

Supplementary Figure 6: OCR level was evaluated in HCT116 and RKO cells with or without ALDOC knockdown

Supplementary Table 1: Primers used in qPCR

Supplementary Table 2: Antibodies used in WB

Supplementary Table 3: Relationship between ALDOC expression and tumor characteristics in patients with colorectal cancer analyzed by Spearman rank correlation analysis

## Data Availability

No datasets were generated or analysed during the current study.
