# Supplementary figures and images for: ALDOC and PGK1 coordinately induce glucose metabolism reprogramming and promote development of colorectal cancer
Source: Mol Med. 2025 Jun 15;31:239. doi: 10.1186/s10020-025-01252-z (PMC12168302; doi:10.1186/s10020-025-01252-z)

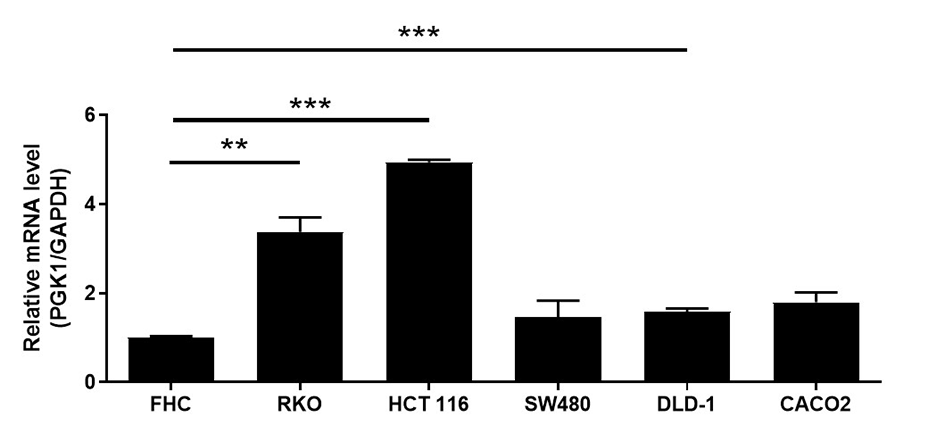

Supplement: Supplementary file 1 — Supplementary Figure 1: The knockdown efficiencies of 3 shRNAs prepared for silencing ALDOC were assessed by qPCR in RKO cells. Data were shown as mean with standard deviation. Data were drawn as mean α SD (n ≥ 3). ** P < 0.01 [file 10020_2025_1252_MOESM1_ESM.png]

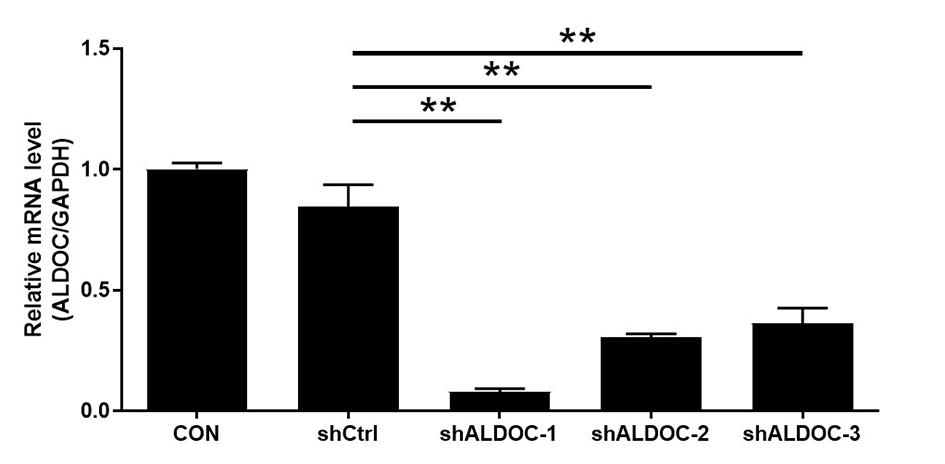

Supplement: Supplementary file 2 — Supplementary Figure 2: Flow cytometry was performed in shCtrl and shALDOC cells for examining cell cycle. Data were drawn as mean α SD (n ≥ 3). ** P < 0.01, *** P < 0.001 [file 10020_2025_1252_MOESM2_ESM.png]

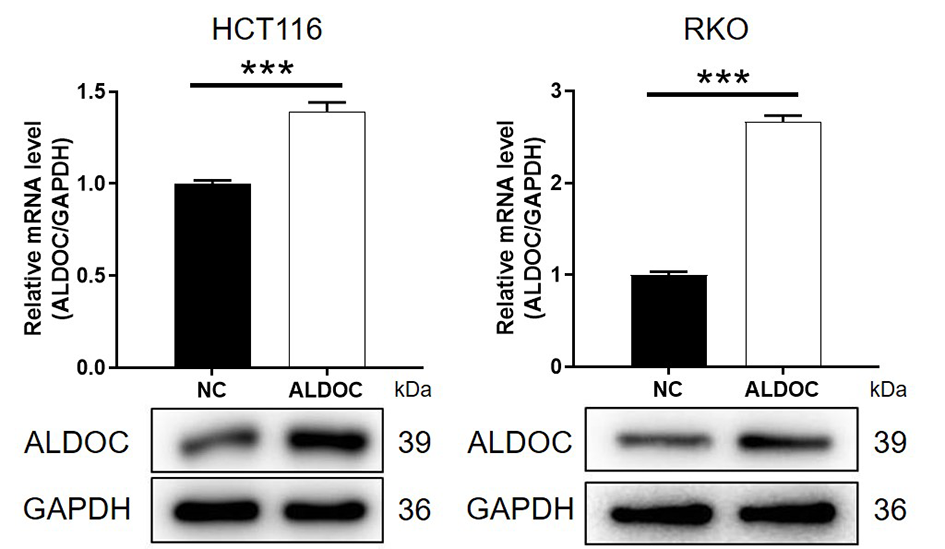

Supplement: Supplementary file 3 — Supplementary Figure 3: The endogenous expression of PGK1 was detected by qPCR in human normal intestinal epithelial cell line FHC and several CRC cell lines including HCT116, RKO, SW480, DLD-1, and CACO2. Data were drawn as mean α SD (n ≥ 3). ** P < 0.01, *** P < 0.001 [file 10020_2025_1252_MOESM3_ESM.png]

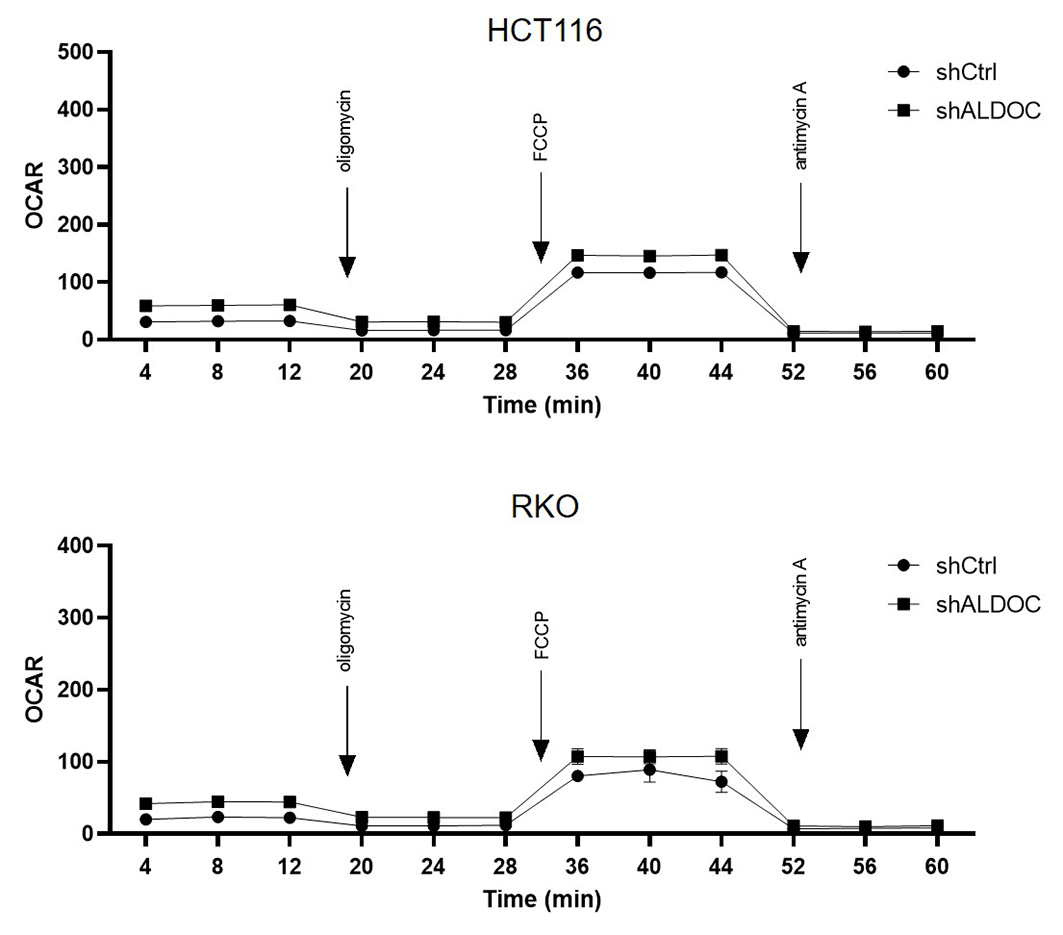

Supplement: Supplementary file 4 — Supplementary Figure 4: The overexpression efficiencies of ALDOC in HCT116 and RKO cells were evaluated based on mRNA and protein levels. Data were drawn as mean α SD (n ≥ 3). *** P < 0.001 [file 10020_2025_1252_MOESM4_ESM.png]

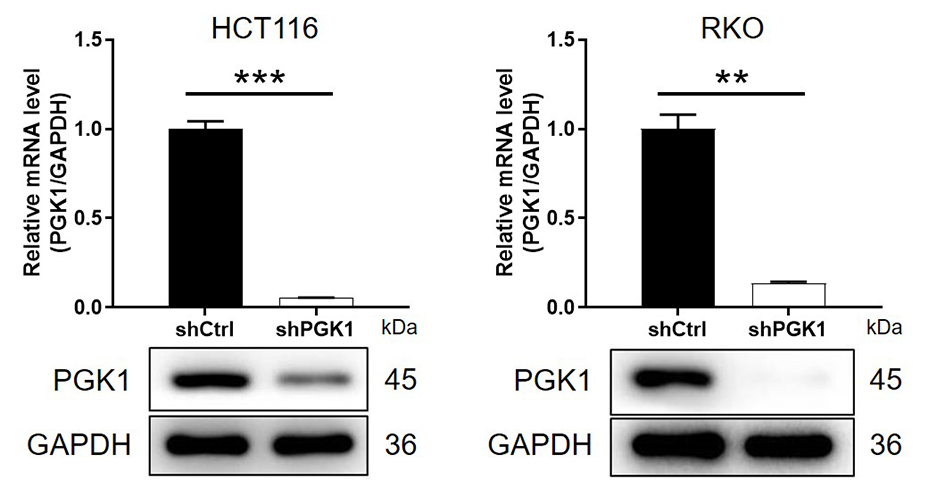

Supplement: Supplementary file 5 — Supplementary Figure 5: The knockdown efficiencies of PGK1 in HCT116 and RKO cells were evaluated based on mRNA and protein levels. Data were drawn as mean α SD (n ≥ 3). ** P < 0.01, *** P < 0.001 [file 10020_2025_1252_MOESM5_ESM.png]

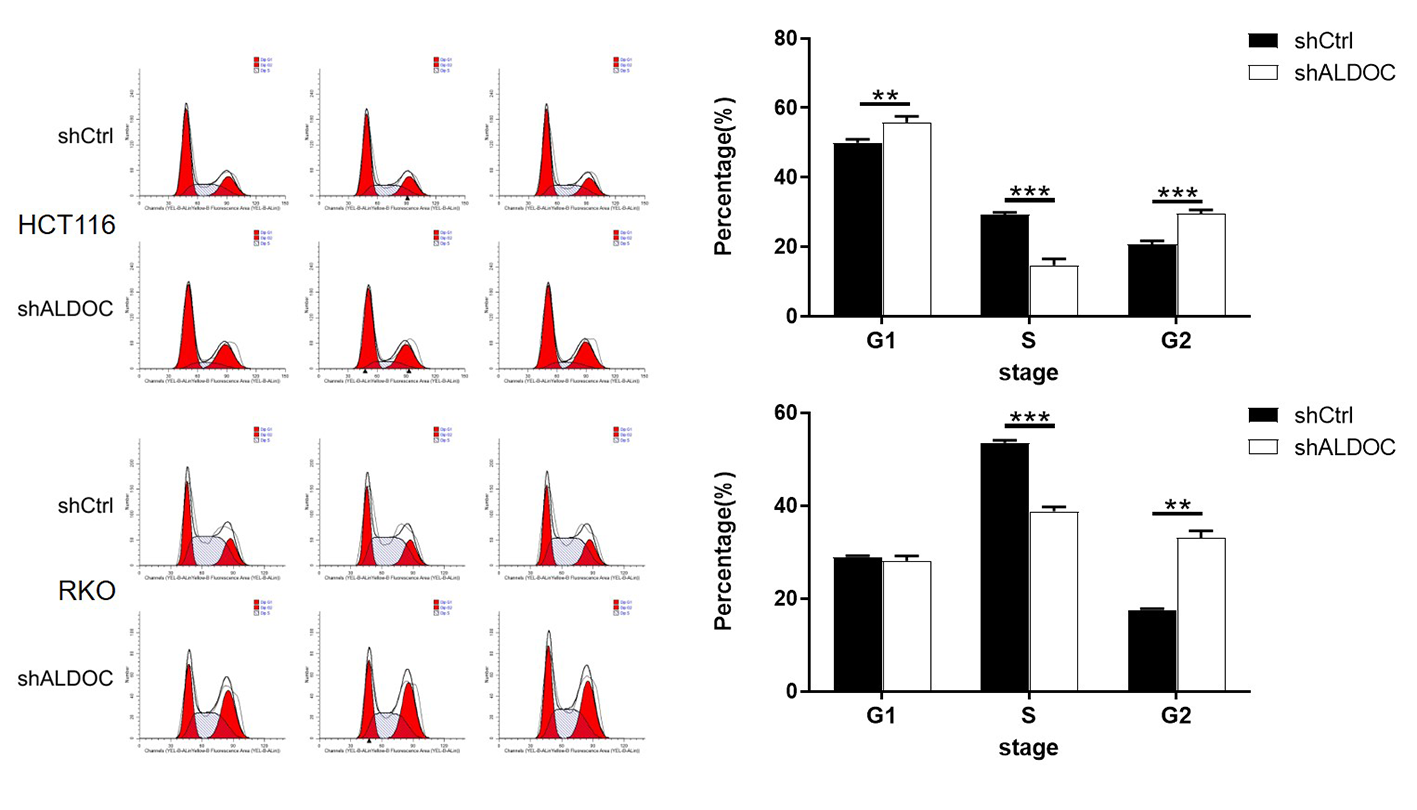

Supplement: Supplementary file 6 — Supplementary Figure 6: OCR level was evaluated in HCT116 and RKO cells with or without ALDOC knockdown [file 10020_2025_1252_MOESM6_ESM.png]
