# Supplementary material for: ALDOC and PGK1 coordinately induce glucose metabolism reprogramming and promote development of colorectal cancer
Source: Mol Med. 2025 Jun 15;31:239. doi: 10.1186/s10020-025-01252-z (PMC12168302; doi:10.1186/s10020-025-01252-z)
Supplement: Supplementary file 7 — Supplementary Table 1: Primers used in qPCR [file 10020_2025_1252_MOESM7_ESM.docx]

Table S1 Primers used in qPCR

| Gene | Forward primer sequence (5’-3’) | Reverse primer sequence (5’-3’) |
| --- | --- | --- |
| GAPDH | TGACTTCAACAGCGACACCCA | CACCCTGTTGCTGTAGCCAAA |
| ALDOC | TACCCCAGAGGAGATTGCCAT | GCCTCTTCTTCGCTCTGACC |
| PGK1 | TGCTGGGCAAGGATGTTCTG | CCACATGAAAGCGGAGGTTCT |
| BNIP3L | ATGTCGTCCCACCTAGTCGAG | TGAGGATGGTACGTGTTCCAG |
| PFKFB4 | TCCCCACGGGAATTGACAC | GGGCACACCAATCCAGTTCA |
| MAP1A | GCTGAGTTCTCCGAGTATGTCT | TGAGTAGCACCGAGTCAATGC |
| ATP6V1G2 | CAGTCAGTCCCAAGGTATCCA | CTGCGGTATTGCTCCACCTC |
| HILPDA | AAGCATGTGTTGAACCTCTACC | TGTGTTGGCTAGTTGGCTTCT |
| SYT11 | ACCAATATCCGACCTAGCTTTGA | TCTGGGTATATGCTGATGCCTT |
| VKORC1 | ATCAATCCAACAGCATATTCGGT | GTCCGCAGGCAACCTAACA |
| CADM2 | AAGAGGATGCAAATCGCAAGA | CACTCCATCATCACTCCGGT |
| WDR54 | AGGGTCCCAACATTGTACTGA | ACAGCAAGCCTGAGTCATCTG |
| CTNND2 | GAGCCCCGGCTTAAACACC | CCTGTTCTTTGACTGAGGCGA |
| DNM3 | TGGAATAACTAAAGTGCCTGTGGG | GCAAGATCAGTGTTGGCTGGA |
