# Supplementary material for: ALDOC and PGK1 coordinately induce glucose metabolism reprogramming and promote development of colorectal cancer
Source: Mol Med. 2025 Jun 15;31:239. doi: 10.1186/s10020-025-01252-z (PMC12168302; doi:10.1186/s10020-025-01252-z)
Supplement: Supplementary file 8 — Supplementary Table 2: Antibodies used in WB [file 10020_2025_1252_MOESM8_ESM.docx]

Table S2 Antibodies used in WB

| Primary antibodies | Dilution in WB | Source species | Company | Catalog No. |
| --- | --- | --- | --- | --- |
| ALDOC | 1:5000 | Rabbit | Proteintech | 14884-1-AP |
| GAPDH | 1:3000 | Rabbit | Bioworld | AP0063 |
| HIF1A | 1:50/1:2000 | Rabbit | Proteintech | 20960-1-AP |
| Histone H3 | 1:2000 | Rabbit | CST | 4499S |
|  |  |  |  |  |
| Secondary antibody | Dilution |  | Company | Catalog No. |
| HRP Goat Anti-Rabbit IgG (WB) | 1:3000 |  | Beyotime | A0208 |
| HRP Goat Anti-Mouse IgG (WB) | 1:3000 |  | Beyotime | A0216 |
