# Supplementary material for: ALDOC and PGK1 coordinately induce glucose metabolism reprogramming and promote development of colorectal cancer
Source: Mol Med. 2025 Jun 15;31:239. doi: 10.1186/s10020-025-01252-z (PMC12168302; doi:10.1186/s10020-025-01252-z)
Supplement: Supplementary file 9 — Supplementary Table 3: Relationship between ALDOC expression and tumor characteristics in patients with colorectal cancer analyzed by Spearman rank correlation analysis [file 10020_2025_1252_MOESM9_ESM.docx]

Table S3 Relationship between ALDOC expression and tumor characteristics in patients with colorectal cancer analyzed by Spearman rank correlation analysis

| Tumor characteristics | index |  |
| --- | --- | --- |
| Lymph node invasion | Spearman correlation | 0.335 |
|  | Significance (two tailed) | 0.001 |
|  | n | 98 |
| [lymphatic](D:/360%E5%AE%89%E5%85%A8%E6%B5%8F%E8%A7%88%E5%99%A8%E4%B8%8B%E8%BD%BD/Dict/8.4.0.0/resultui/html/index.html#/javascript:;) [metastasis](D:/360%E5%AE%89%E5%85%A8%E6%B5%8F%E8%A7%88%E5%99%A8%E4%B8%8B%E8%BD%BD/Dict/8.4.0.0/resultui/html/index.html#/javascript:;) (N) | Spearman correlation | 0.327 |
|  | Significance (two tailed) | 0.001 |
|  | n | 98 |
| T [Infiltrate](D:/360%E5%AE%89%E5%85%A8%E6%B5%8F%E8%A7%88%E5%99%A8%E4%B8%8B%E8%BD%BD/Dict/8.4.0.0/resultui/html/index.html#/javascript:;) | Spearman correlation | 0.292 |
|  | Significance (two tailed) | 0.004 |
|  | n | 98 |
| Stage | Spearman correlation | 0.448 |
|  | Significance (two tailed) | <0.001 |
|  | n | 98 |
